# Viral burden is associated with age, vaccination, and viral variant in a population-representative study of SARS-CoV-2 that accounts for time-since-infection-related sampling bias

**DOI:** 10.1371/journal.ppat.1011461

**Published:** 2023-08-14

**Authors:** Helen R. Fryer, Tanya Golubchik, Matthew Hall, Christophe Fraser, Robert Hinch, Luca Ferretti, Laura Thomson, Anel Nurtay, Lorenzo Pellis, Thomas House, George MacIntyre-Cockett, Amy Trebes, David Buck, Paolo Piazza, Angie Green, Lorne J Lonie, Darren Smith, Matthew Bashton, Matthew Crown, Andrew Nelson, Clare M. McCann, Mohammed Adnan Tariq, Claire J. Elstob, Rui Nunes Dos Santos, Zack Richards, Xin Xhang, Joseph Hawley, Mark R. Lee, Priscilla Carrillo-Barragan, Isobel Chapman, Sarah Harthern-Flint, The COVID-19 Genomics UK (COG-UK) consortium, David Bonsall, Katrina A. Lythgoe

**Affiliations:** 1 Big Data Institute, Nuffield Department of Medicine, University of Oxford, Old Road Campus, Oxford, United Kingdom; 2 Sydney Infectious Diseases Institute (Sydney ID), School of Medical Sciences, Faculty of Medicine and Health, University of Sydney, Sydney, Australia; 3 Pandemic Sciences Institute, University of Oxford, Old Road Campus, Oxford, United Kingdom; 4 Department of Mathematics, University of Manchester, Manchester, United Kingdom; 5 The Alan Turing Institute, London, United Kingdom; 6 Wellcome Centre for Human Genetics, Oxford, United Kingdom; 7 The Hub for Biotechnology in the Built Environment, Department of Applied Sciences, Faculty of Health and Life Sciences, Northumbria University, Newcastle upon Tyne, United Kingdom; 8 Department of Applied Sciences, Faculty of Health and Life Sciences, Northumbria University, Newcastle upon Tyne, United Kingdom; 9 Department of Biology, University of Oxford, Oxford, United Kingdom; Tel Aviv University, ISRAEL

## Abstract

In this study, we evaluated the impact of viral variant, in addition to other variables, on within-host viral burden, by analysing cycle threshold (Ct) values derived from nose and throat swabs, collected as part of the UK COVID-19 Infection Survey. Because viral burden distributions determined from community survey data can be biased due to the impact of variant epidemiology on the time-since-infection of samples, we developed a method to explicitly adjust observed Ct value distributions to account for the expected bias. By analysing the adjusted Ct values using partial least squares regression, we found that among unvaccinated individuals with no known prior exposure, viral burden was 44% lower among Alpha variant infections, compared to those with the predecessor strain, B.1.177. Vaccination reduced viral burden by 67%, and among vaccinated individuals, viral burden was 286% higher among Delta variant, compared to Alpha variant, infections. In addition, viral burden increased by 17% for every 10-year age increment of the infected individual. In summary, within-host viral burden increases with age, is reduced by vaccination, and is influenced by the interplay of vaccination status and viral variant.

## Author summary

The SARS-CoV-2 epidemic in the United Kingdom (UK) has been characterized by the successive transmission of distinct viral variants. Viral variation can impact viral properties, including infectiousness and pathogenicity. These properties may be linked to the amount of virus present in an infected individual. In this study, we examined the association between intra-patient viral burden and a range of factors, including viral variant, using a large, population-representative SARS-CoV-2 survey conducted in the UK. As part of our investigation, we developed a novel method to account for bias in sampled viral burden resulting from the study’s sampling methodology. Our findings indicate that viral burden within the host increases with age, is reduced by vaccination, and is influenced by the interplay between vaccination status and viral variant.

## Introduction

The SARS-CoV-2 epidemic in the United Kingdom (UK) has been characterised by the appearance of a series of distinct viral variants that, in order of emergence, include the B.1.177 lineage, and the Alpha (B.1.1.7 lineage), Delta (B.1.617.2 lineage) and Omicron (BA.1, BA.2, BA.4 and BA.5 lineages) variants. Explaining their successive abilities to spread, the Alpha, Delta and Omicron variants have been estimated to have a transmission advantage of 43–100% [1–3], 60–70% [4] and 52% [5] compared to their preceding variant. The underlying causes of these differences are unclear, but could include differences in within-host viral burden [6], infectious period, or the per-virion probability of between-host transmission. In turn, these could be influenced by many factors [7], including changes in virus attachment to human cells and the continuous interplay of population acquisition of immunity and the emergence of immune escape variants [8, 9]. In this study, we investigate the association between within-host viral burden and viral variant by analysing nose and throat swabs collected as part of the UK’s nationally representative SARS-CoV-2 surveillance study [10, 11].

A number of studies have compared viral burden amongst infections with the Alpha variant and predecessor variants (S1 Table)[12–18] with mixed findings. For example, two detailed longitudinal surveys of a small number of infected individuals have suggested that viral burden is similar among infections with the Alpha variant and predecessor strains [16, 17]. However, a much larger, but less intensive study of viral burden at symptom onset has identified that viral burden is higher among infections with the Alpha-variant, compared to those with a predecessor lineage [15]. The impact of later variants on viral burden has also been studied [11, 15, 16, 19], indicating that viral burden is higher among Delta-variant, compared to the Alpha-variant infections, in vaccinated individuals [11] in one survey, but that there is no difference between infections with these variants in another [16]. The study design and cohorts used to investigate viral burden have varied and this may explain the different findings. In addition to the differences in sample sizes and sampling frequency, the study populations have varied. Some have been based upon testing symptomatic individuals or their close contacts [12, 14, 15] and have thereby excluded some asymptomatically infected individuals, who make up an estimated 40% [20] of infections. Others have focussed on a specific group of people, with examples being hospitalized individuals [12] and persons associated with a professional sporting league [16]. Methods to identify variants have also varied, with some surveys using Spike gene target failure (SGTF) [12, 14, 15] during PCR testing or sample date [11] to classify the viral variants, whereas other have used whole genome sequencing [13, 16, 17].

The Office for National Statistics (ONS) COVID-19 Infection Survey (CIS) is a large household-based surveillance study based in the United Kingdom [10, 11]. We analysed data from the CIS to investigate the impact of viral variant on viral burden. The survey randomly selects private households on a continuous basis from address lists and previous surveys to provide a representative UK sample. Individuals were asked to provide information that included demographics, symptoms, and vaccination details. As part of the survey, nose and throat swabs were collected and tested for SARS-CoV-2 using RT-PCR, and, if positive, individuals with a cycle threshold (Ct) less than 30 were sequenced using whole genome sequencing. Since the Ct value of a sample is inversely correlated with log_10_(viral burden) of that sample [21], this study design enables viral burden to be investigated. Although the accuracy with which the viral burden sampled from a nose and throat swab informs the viral burden throughout the body is unclear [22], this study does allow for investigation into viral burden in a manner that avoids biases associated with samples from symptomatic individuals or small studies of particular demographic groups.

The survey is simultaneously a cross-sectional survey of the population through time and a longitudinal survey of individuals, with individuals sampled approximately weekly during the first month following enrolment and then monthly thereafter, regardless of symptoms. This weekly or monthly sampling leads to uncertainty in the time-since-infection of positive samples. In addition, the different epidemiological trajectories of the variants mean that the distribution of time-since-infection for each variant at any given time can be skewed depending on when the samples were collected. For example, if a variant is increasing in prevalence, a cross sectional sample will contain more individuals with that variant who are earlier on in their infection, compared to those who are later on in their infection [23]. Because within-host viral burden trajectories are asymmetric, with the peak in viral load closer to the start of infection than to the end [16], this can affect the sampled distribution of viral burden and complicate comparisons between viral variants. The impact of SARS-CoV-2 epidemiology on sampled Ct values is sufficiently strong for its shifts to be inferred from changes in Ct values measured over time [23, 24].

We are unaware of any published studies comparing viral burden associated with viral variants from a large population-representative surveillance survey that directly estimates the impact of variant-specific epidemiological trajectories. Here, we address this gap by developing a methodology that directly estimates the combined impact of variant-specific within-host viral burden and epidemiological trajectories on randomly sampled viral burden. We apply this methodology to data from the CIS to investigate the impact of a range of factors, including variant, vaccination status, and age, on viral burden, as measured by Ct values. As many countries move towards implementing SARS-CoV-2 surveillance surveys, the concepts and methodologies described here will be valuable for informing public health decisions. Moreover, the concepts and methods will be applicable to other pathogens for which sampled viral burden is affected by epidemiological dynamics.

## Results

### Covid-19-Infection survey

We used data from the Office for National Statistics Covid infection survey (ISRCTN21086382CT, https://www.ndm.ox.ac.uk/covid-19/covid-19-infection-survey). The survey has been described in detail elsewhere [24] and the survey protocol is provided online (https://www.ndm.ox.ac.uk/covid-19/covid-19-infection-survey/protocol-and-information-sheets). However, in brief, private households were randomly selected on a continuing basis in order to provide a representative sample of inhabitants of the UK. Following agreement to participate, self-collected nose and throat swabs were taken by participants–or their parents/guardians if they were aged 12 or under–as instructed by a study worker. The intended schedule of swabbing was weekly for the first month of participation and monthly thereafter, for up to a year. However, there was variability among participants due to missed or late swabs, and participants could also choose to participate only once, or only for the first month, rather than on an ongoing basis, and were also free to leave the study at any time. Overall however, attrition rates for the survey were low (typically less than 1% of participants per month), as detailed in a report by the ONS [25], providing confidence in the accuracy of the survey’s findings.

Individuals were asked about demographics, symptoms, contacts, and relevant behaviours (https://www.ndm.ox.ac.uk/covid-19/covid-19-infection-survey/case-record-forms). An analysis of how representative the Covid-19 Infection Survey is of the UK population has previously been described in detail [26]. In a random 10–20% of households, participants who were 16 years or older were invited to provide monthly venous blood samples for assays of anti-trimeric spike protein IgG.

### RT-PCR Covid-19 testing

In this study we focused on swabs and blood samples that were part of the CIS and were sent (at ambient temperatures) to the UK’s national Lighthouse Laboratory at Glasgow. RT-PCR for three SARS-CoV-2 genes (N protein, S protein, and ORF1ab) used the Thermo Fisher TaqPath RT-PCR COVID-19 Kit, analysed using UgenTec Fast Finder 3.300.5 (TaqMan 2019-nCoV Assay Kit V2 UK NHS ABI 7500 v2.1). The Assay Plugin was used to convert the qualitative amplification Assay PCR raw data from the ABI 7500 Fast into test results with minimal manual intervention, using an assay-specific algorithm and decision mechanism. Samples were considered positive if at least one N gene and/or ORF1ab gene was present. The lighthouse laboratory was assessed by the external quality assessment laboratories, NEQAS. We analysed RT-qPCR SARS-CoV-2 positive samples that were sequenced at Oxford University (sampled between 27/09/20 and 17/06/21) or Northumbria University (sampled between 20/09/21 and 19/01/22) and had a Ct≤30. These samples cover the period of the epidemic that includes sections of the B.1.177, Alpha, Delta, and BA.1 Omicron waves.

### Sequencing and lineage identification

All swabs were tested for SARS-CoV-2 using RT-QPCR, and the cycle threshold (Ct) values of positive samples were recorded. For samples collected before December 2020, real-time sequencing of samples was undertaken where possible, with some additional retrospective sequencing. From December 2020 onwards, sequencing was attempted on all positive samples with Ct≤30. Sequenced samples collected between 27^th^ Sep 2020 and 17^th^ July 2021 were sequenced at the University of Oxford using veSEQ. This employs an RNASeq protocol based on a quantitative targeted enrichment strategy [27] and sequencing on the Illumina Novaseq platform. For a full description of the sequencing protocol, see [27, 28]. Samples collected between 20^th^ Sep 2021 and 19^th^ Jan 2022 were sequenced at the University of Northumbria using the CoronaHiT [29] variant of the ARTIC protocol and Illumina Novaseq 550.

All samples sequenced in Oxford with Ct≤30 were retained for analysis, with the added restriction of ≥50% genome coverage required for samples sequenced in Northumbria. For the periods that these two datasets span, the number of positive samples tested in Glasgow was 51101. Amongst these samples 32852 (64%) had a Ct≤30. Consensus sequences were produced using the *shiver* pipeline [30] and lineages were assigned using PangoLEARN [31], with samples assigned as B.1.177 (and sublineages), Alpha (B.1.1.7 and sublineages), Delta (B.1.617.2 and sublineages) and BA.1 Omicron (BA.1 and sublineages) used for this analysis. For Oxford sequenced samples with <50% coverage, and which could not be reliably assigned using PangoLEARN, we assigned one of the four major lineages if a consensus base was called at three or more lineage defining sites, and with more than two-thirds of these calls consistent with the lineage. Of a total of 5562 and 27290 samples with a Ct≤30 from the time periods coinciding with when samples were sent for sequencing at Oxford and Northumbria, a lineage could be assigned for 5315 (96%) and 21805 (80%) samples, respectively. Among these samples 4829 (91%) and 20191 (93%) could reliably be assigned to one of the four lineages described above, and the remainder were excluded from further analysis. Additional data processing steps are detailed in the Methods section. Because the sequencing protocols and data availability criteria used at Oxford and Northumbria were different, data from the two sequencing labs were incorporated into separate regression analyses.

### Serology testing

Blood samples were analyzed for antibodies at the University of Oxford. The immunoassay used for this purpose was developed by the University of Oxford’s National SARS-CoV-2 Serology Assay Evaluation Group in conjunction with the study team. The study protocol was approved by the South Central Berkshire B Research Ethics Committee (20/SC/0195). A positive result was recorded for antibody titres above 8 million units (National SARS-CoV-2 Serology Assay Evaluation Group, 2020) on the original fluorometric version of the assay, and above 42 units on the colorimetric version used from March 1, 2021.

### A new framework to infer epidemiologically adjusted Ct values

**Fig 1:**
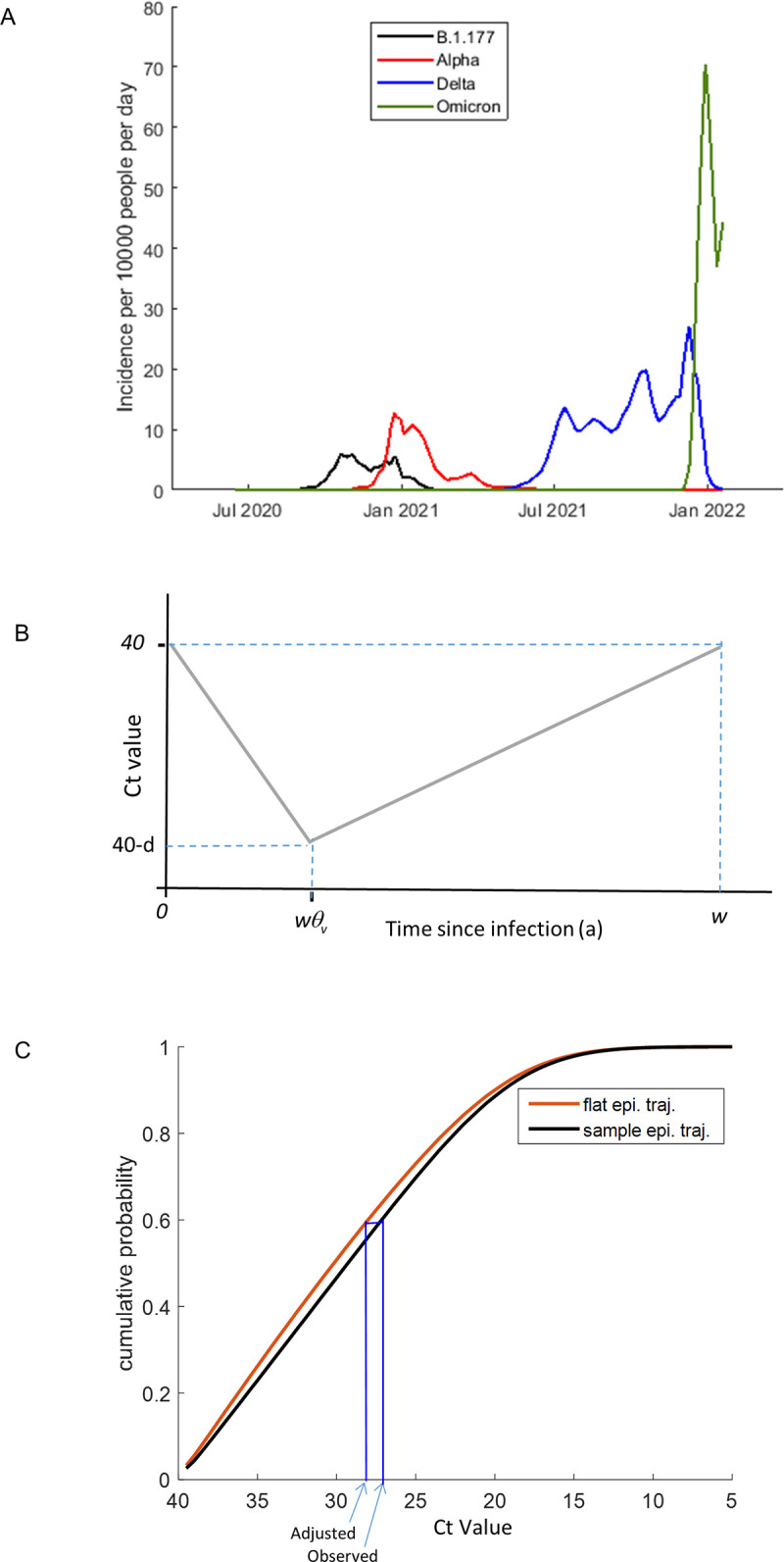
A method for estimating epidemiologically adjusted Ct values. A) Inferred daily incidence with the B.1.177 lineage and the Alpha, Delta and BA.1 Omicron variants between July 2020 and January 2022 in the UK. These were estimated to equal the product of the daily incidence of SARS-CoV-2 and the fraction of incident infections of that variant. B) Within-host Ct trajectories were assumed to be valley shaped, with infected period (width) *w*, and depth *d*. The valley trough was estimated to be a fraction *θ*_*v*_ across the width. C) Adjusted Ct values were inferred by first estimating the cumulative probability distribution of Ct values based upon the sample date and the known epidemiological trajectory of the sample variant and identifying the percentile at which the observed Ct value falls within this distribution. Second, the cumulative probability distribution of Ct values under an assumption of a flat epidemiological trajectory was estimated and the Ct value at the selected percentile was identified.

To enable us to investigate the association between viral burden and viral variant, we developed a framework that adjusts observed Ct values to account for the different epidemiological trajectories of different viral variants (see methods). In brief, variant-specific incidence rates for each of the major variants in the sample data (B.1.177, Alpha, Delta and BA.1 Omicron) (Fig 1A) were inferred by combining estimates of total SARS-CoV-2 incidence rates in England (www.ons.gov.uk/peoplepopulationandcommunity/healthandsocialcare/conditionsanddiseases/datasets/coronaviruscovid19infectionsurveydata) with estimates of the proportion of incident infections with each variant, as inferred from the COVID-19 infection consortium data repository (www.cogconsortium.uk/). These data were used rather than the equivalent estimates available directly from the CIS to prevent the introduction of a time lag between incidence and prevalence into our study. The variant-specific incidence rates were combined with normally distributed infection periods to estimate how the expected distribution of time since infection from randomly sampled individuals changes over calendar time for each of the variants. For each PCR positive sequenced sample in our analysis, the expected distribution of the time since infection corresponding to its variant and sample date was identified and truncated to account for expected bounds, where these could be determined by previous positive or negative samples from the same individual. For each sample, we next estimated an expected distribution of Ct values. This was achieved by assuming that within-host Ct values are described by a piecewise, valley-shaped trajectory (Fig 1B) with depth (viral burden peak) and width (infected period) taken from normal distributions. The timing of the valley trough (peak viral burden) was fixed at a chosen fraction across the width. The parameters describing these metrics were estimated from an alternative data source [16]. However, the mean maximum valley depth (mean peak viral burden) was iteratively inferred, and other parameters–including the timing of the peak viral burden–were varied during sensitivity analyses. For each sample, an adjusted Ct value was then inferred as follows, and as demonstrated graphically in Fig 1C. Firstly, we identified what percentile the observed Ct value lies at among the Ct distribution that is expected, based upon the sample variant (see the black line in Fig 1C). Secondly, we selected the Ct value at the corresponding percentile, from the Ct distribution that is expected, based upon a flat (constant incidence) epidemic trajectory (see the red line in Fig 1C).

### Ct values from early and late during the Alpha wave are more closely aligned after epidemiological adjustment

**Fig 2:**
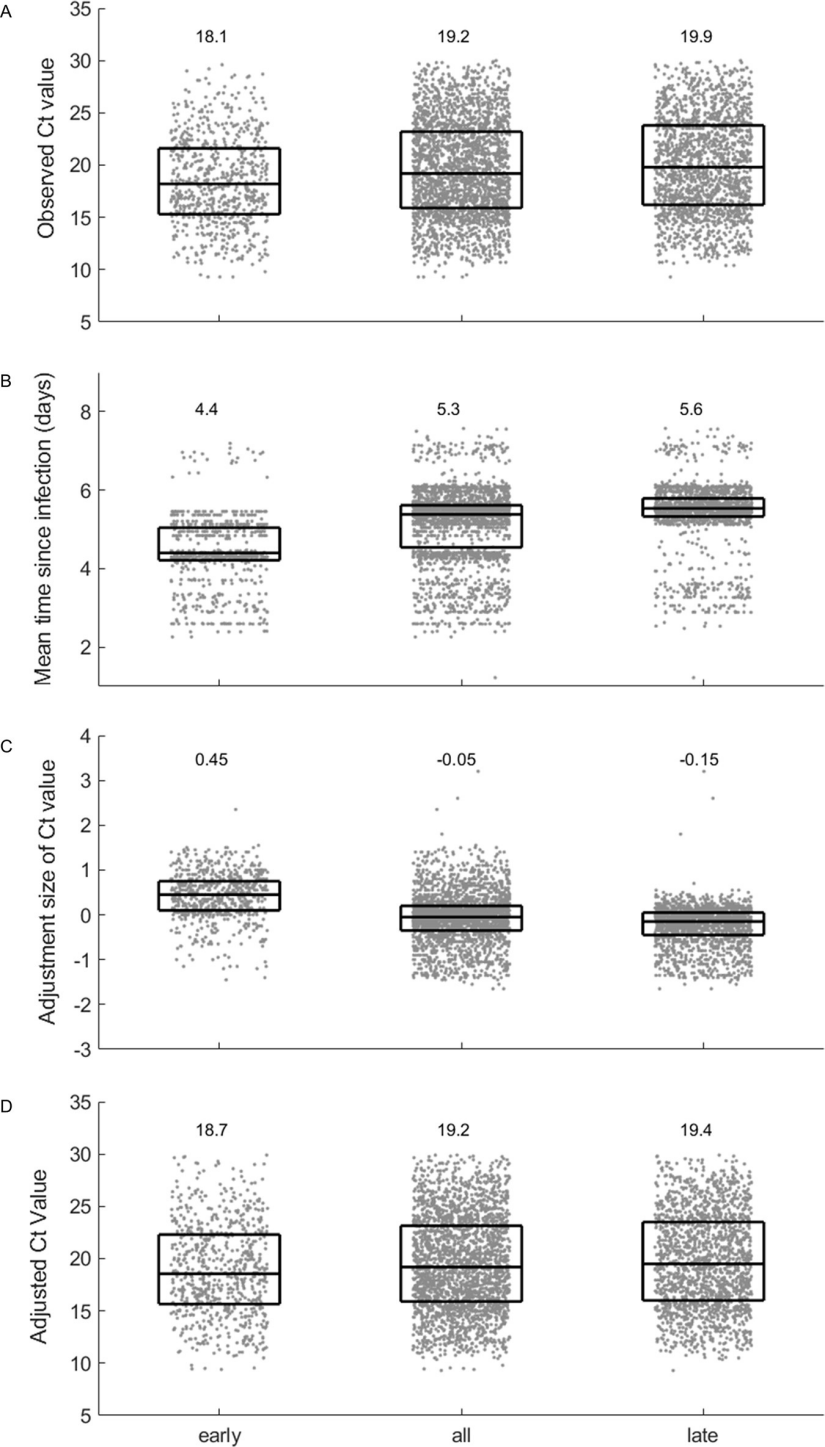
Epidemiological adjustment results in more closely aligned estimates of mean viral burden from samples taken early and late during the Alpha wave. Samples that correspond to Alpha-variant infections in individuals who were unvaccinated and had not been identified as having had a prior exposure were split according to sample date. Four metrics were applied to data from the early phase, all phases and the late phase. In each panel, median and interquartile ranges are overlaid onto individual data points (equivalent distribution plots are provided in S2 Fig). A) The observed Ct values are, on average, higher for late phase, compared to early phase samples. B) The estimated mean time since infection is, on average, longer for late-phase, compared to early-phase samples. C) The Ct adjustment size is, on average, positive for early phase samples, negative for late phase samples and negligible when all data are considered. D) On average, the adjusted Ct values relating to the early and late phase are more closely aligned than the observed Ct values. However, adjusted values remain, on average, higher in late-phase, compared to early-phase samples.

Since we had data spanning a large portion of the epidemiological trajectory of the Alpha wave in the UK, we determined the impact of our method when applied to data collected at different stages during its trajectory. We applied the adjustment to Alpha-variant samples collected from unvaccinated individuals who had no known prior exposure (i.e. no recorded prior infection nor a positive spike-antibody test) (n = 2465; approximately 15% of individuals prior to the start of the Alpha wave were tested for antibodies). By splitting the samples according to sample date into sets representing the early-phase (growth) and late-phase (decline) of the Alpha wave, we visualised how the timing of sampling during the epidemiological trajectory impacted observed Ct values (Fig 2).

The median of the unadjusted Ct values was lower for early-phase samples than for late-phase samples (Fig 2A), consistent with the expected impact of the epidemiological effect. For each sample, our method estimates a probability distribution for the time since infection for that sample, based upon the sample variant, sample collection date, and, where available, the dates of recent positive and negative samples within the same infection. The mean time since infection derived from each of these distributions is plotted in Fig 2B. On average the mean time since infection is longer among the late-phase compared to early-phase samples. Because Ct values are, on average, lower in early infection compared to late infection (Fig 1B), the adjustment acted in the opposite direction and increased the Ct values of early-phase samples, but decreased the Ct values of late-phase samples (Fig 2C).

When the epidemiological adjustment was applied to the Ct values, the adjusted distribution of Ct values for the early-phase and late-phase were more closely aligned compared to the unadjusted values (Fig 2D). For comparison, the application of the method to data from the whole Alpha wave is also shown (middle column in Fig 2), revealing that the net adjustment applied to the full set of samples is negligible. This emphasises the value of using the epidemiological adjustment when samples are only available for part of the epidemiological trajectory of a variant, such as during the emergence phase of a new variant.

### The asymmetry of the within-host viral trajectory impacts comparisons

**Fig 3:**
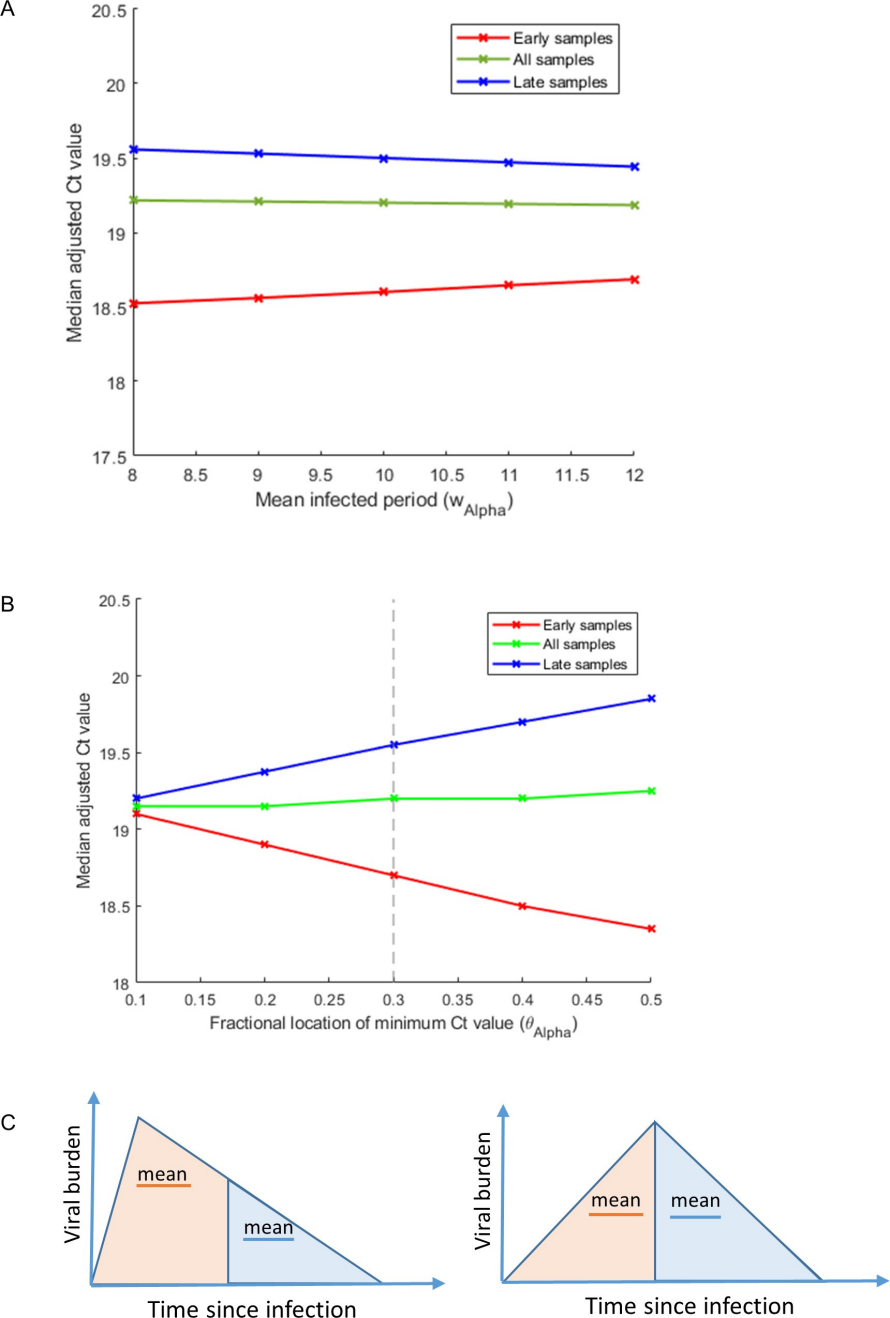
The epidemiological stage and the asymmetry of the within-host viral trajectory impact the Ct adjustment size. In panels A) and B) samples that correspond to Alpha-variant infections in individuals who were unvaccinated and had not been identified as having had a prior exposure are split according to sample date. The medians of the adjusted Ct values are plotted for early samples (red), late samples (blue) and all samples (green) under different assumptions about the asymmetry and the mean width of the within-host viral burden trajectory. In panel A) the infected period is varied under an assumption that the viral burden trajectory is skewed towards the start of infection (*θ*_Alpha_ = 0.3). This shows that Ct values are lower (viral burden is higher) amongst samples taken earlier on during infection, but vary to only a limited degree with changes in the mean infected period (*w*_Alpha_). In panel B) the fractional location of the peak viral burden, *θ*_Alpha_, is varied under the assumption that the mean infected period (with Ct value ≤40) is 10 days (*w*_Alpha_ = 10) [16]. This shows that the asymmetry of the within-host viral burden trajectory measurably impacts the adjusted Ct values and that the early- and late-phase Alpha-variant samples are most closely aligned when *θ*_Alpha_ is smaller. Panel C) highlights how, when the within-host trajectory is skewed towards earlier during infection, viral burden sampled during early infection will on average be higher than that sampled later on in infection.

Our framework highlights that the combined impact of the shape of the within-host viral trajectory and the epidemiological stage of a variant can affect viral burden measured at the population level. Plausible changes to our assumption of the mean infected period have only a small impact upon the adjusted values (Fig 3A), whereas plausible changes to the fractional position of the viral burden peak across this period have a bigger effect on the adjusted values (Fig 3B) (although absolute changes are still modest compared with variability between individuals). The closer the peak viral burden is to the start of infection, the greater the epidemiological correction applied to samples selected from just early on or just late on during the Alpha wave. This can be understood by noting that in a random sample, early-phase samples have, on-average, shorter times since infection than late-phase samples and the greater the asymmetry of the within-host viral burden, the greater the difference in expected viral burden between infections in the earlier or later phases of infection (Fig 3C).

In calculating the adjusted Ct values for samples with the Alpha-variant (Fig 2D) we assumed that peak viral burden occurs at a fraction 0.3 across the infected period, based upon prior data from 103 individuals [16]. It is noteworthy that using this parameter estimate, the median adjusted Ct value remains higher for late-phase, compared to early-phase samples. This can be visualised by comparing the red and blues lines shown in Fig 3B at a value *θ*_Alpha_ = 0.3 along the x-axis (grey dashed vertical line). For a mean infected period of 10 days [16] (classified according to having a Ct value above 40), this would correspond to the peak infection occurring at day 3. The median adjusted Ct values of the early-phase and late-phase samples are closer when the asymmetry of the within-host trajectory is increased. Arguably, changing this parameter estimate so that the peak is closer to the start of infection than we have assumed, may therefore provide a better estimate of its true value compared to the one that we derived from published work.

However, there are other explanations for a higher viral burden (lower Ct values) in the early-phase samples. Because the CIS conducted a large round of recruitment in September-October 2020, many participants at the start of the Alpha wave were still undergoing more regular–approximately weekly–follow-up, meaning they may genuinely have been sampled closer to the start of infection in the early phase than the later phase. Second, CIS tested antibodies in only ~15% of participants prior to the Alpha wave, so we cannot rule out that some samples come from individuals who had an undetected prior infection and that the number of such individuals increased over the duration of the Alpha wave. For a description of the relationship between sero-status and test positivity see Walker et al. [24]. It is thus credible that more intensive sampling and lower population levels of immunity present earlier on in the Alpha wave could contribute to the pattern of lower adjusted Ct values in early-phase compared to late-phase samples.

### Investigating variables associated with within-host viral burden

We investigated whether variables, including viral variant, are associated with adjusted Ct values sampled in the CIS and sequenced at Oxford or Northumbria Universities using partial least squares regression (PLS). Samples sequenced at Oxford were collected between 27^th^ September 2020 and 17^th^ July 2021, and cover the period of the epidemic that includes parts of the B.1.177, Alpha and Delta waves. Samples sequenced at Northumbria were collected between 20^th^ September 2021 and 19^th^ January 2022 and cover parts of the Delta and BA.1 Omicron waves. We have analysed the samples sequenced from the two centres separately so that differences in sequencing protocols and genomic coverage inclusion criteria do not affect our results.

**Fig 4:**
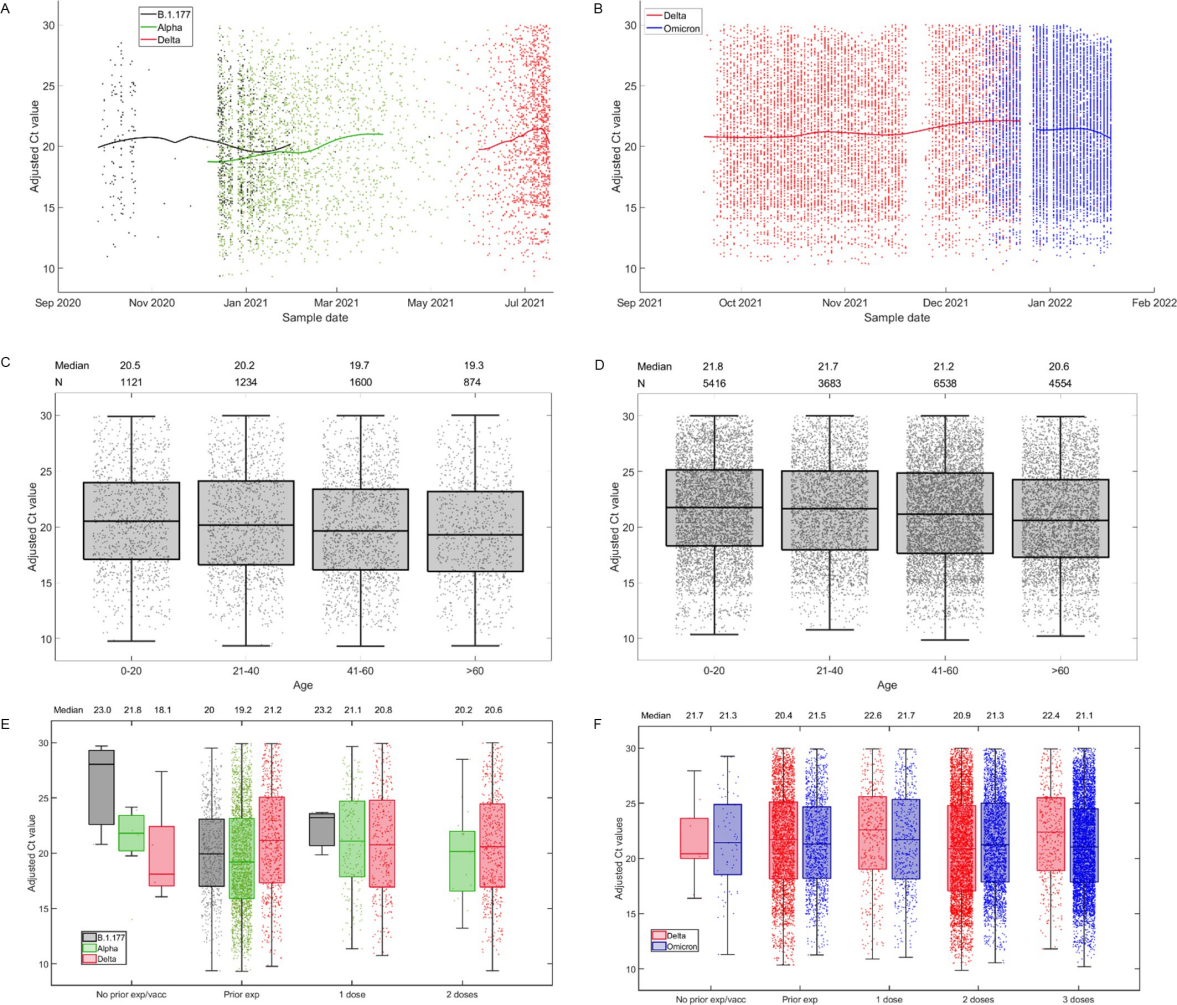
Adjusted Ct values plotted against different factors. For samples sequenced at Oxford (A, C and E) and at Northumbria (d, e and f), adjusted Ct values are plotted against different variabless. Panel A) and B) show a LOESS fit (smoothing parameter = 0.55) of adjusted Ct values over sample date, categorised by variant. Panels C) and D) show box and whisker plots of adjusted Ct values by age category. Panels E) and F) show box and whisker plots of adjusted Ct values by prior vaccination and/or infection, by variant. Horizontal lines represent the median and interquartile range. Parameter values used in these calculations are listed in Table 3.

Adjusted Ct values for samples from these two centres are shown in Fig 4, categorised according to sample date (Fig 4A and 4B), participant age (Fig 4C and 4D), and a combination of prior exposure category and variant (Fig 4C and 4F). Using partial least squares regression analysis, we assessed the impact of sample date, sex, ethnicity, health care worker status, first vaccine dose product (AstraZeneca ChAdOx1 nCoV-19, Pfizer/BioNTech BNT162b2) and prior exposure category (unvaccinated with no known prior exposure, unvaccinated with a known prior exposure, 1 vaccine dose, 2 vaccine doses, 3 vaccine doses) on adjusted Ct values. It is noteworthy that because not all individuals were tested for spike antibodies prior to infection that individuals with no known prior exposure could have experienced an unidentified prior exposure. In addition, within each prior exposure category we assessed the impact of variant (B.1.177, Alpha, Delta and BA.1 Omicron).

PLS was used for this study instead of standard multivariate regression analysis, because it explicitly acknowledges multicollinearity, which is measurable in our data. This was evident from an investigation of variance inflation factors (discussed below) and from the fact that standard regression analyses gave varying results with different selection procedures. Unlike standard regression analysis, PLS does not explicitly test effects for individual explanatory variables. Instead it acknowledges the issue of multicollinearity by analysing latent (principle) components. Whilst the results are not as intuitive as for multivariate regression, PLS does produce Beta scores (which can be considered equivalent to regression coefficients), and variance in projection (VIP) scores, which can be used to assess the magnitude and importance of the contribution of the different variables to the response, respectively. Other methods, such as Bayesian model averaging (BMA) or principle component analysis (PCA) could alternatively have been employed for this problem. However, the results from BMA can be influenced by the choice of prior and the dimension reduction technique in PCA only acknowledges the relationships between the explanatory variables. PLS, however, is independent of user choices and additionally acknowledge the relationships between the explanatory variables and the predictor variable.

Prior to application of the PLS regression model, we investigated multicollinearity among predictor variables by calculating variance inflation factor (VIF) values (S2 Table). Although no clear consensus on VIF threshold criteria exist, a review of relevant literature (summarized in [32]) suggests that a VIF> 3.3 can be considered an indicator of moderate multicollinearity and a VIF>5 an indicator of strong multicollinearity. Because several of the VIF values among both sample sets were greater than 3.3, we analysed our data using PLS regression to acknowledge the difficulties in disentangling the relative roles of different factors in explaining viral burden.

### Viral burden is higher among older individuals

For samples sequenced in Oxford, six components (linear combinations of the predictors that are orthogonal to each other) describe the data (S1A Fig), as determined by the number that minimises the mean squared prediction error. Although these components only explain a small amount of variance in the adjusted Ct values (2.1%), the first two are both significant in predicting the values in a quantile median regression model (p<0.0001 and p = 0.0004) (used to acknowledge non-normality in the residuals). For the Northumbria samples, six latent components also minimise the mean squared prediction error, the first three of which significantly predict (p<0.0001) the adjusted Ct values (S1A and S1B Fig). This analysis highlights that, taken together, factors included in our model significantly impact viral burden. For reference, loading plots for the first two latent components of each sample are shown in S1C and S1D Fig.

Beta scores and variance in projection (VIP) scores, used to assess the magnitude and importance of the contribution of the different variables to the response, respectively, are provided in (Table 1). Variables with VIP values greater than 1 are typically considered to be important and those with VIP values greater than 0.8 are considered to be borderline important (and were retained in our model). Using this approach, we identified sample collection date as an important predictor of Ct values among the samples sequenced at both Oxford (Beta score = 0.008 per year, VIP = 1.83) and Northumbria (Beta score = 0.013 per year, VIP = 1.22). Infection-acquired immunity increased in the population over this period ([33]), which likely contributed to this effect. Age was also an important predictor in both sample sets (Oxford: Beta score = -0.016 per year, VIP = 1.11; Northumbria: Beta score = -0.021 per year, VIP = 1.84). Based upon a 3-point decrease in Ct value being equivalent to a 10-fold increase in viral load (see [34]), these age effects equate to an approximate 14% and 17% increase in viral load for every 10 year age increase, respectively.

There was no strong evidence of an association between viral burden and either sex, ethnicity or being a health care worker (see Table 1 data).

**Table 1:** Beta scores and variance in projection (VIP) values for the partial least squares analysis of samples sequenced in Oxford and Northumbria. A breakdown of sample sizes, by category is also provided. *based upon a Ct value decrease of 3 being equivalent to a 10-fold increase in viral load [34].

Samples	Oxford			Northumbria		
Result	N (4829)	Beta score (viral load factor change*)	VIP	N (20191)	Beta score (viral load factor change)	VIP
Included in model						
**Sample Date**		0.008 per year (0.99 per year)	1.83		0.013 per year (0.99 per year)	1.22
**Age**		-0.016 per year (1.13 per 10 years)	1.11		-0.021 per year (1.17 per 10 years)	1.84
**Prior exposure**						
*Ref = Unvaccinated with no known prior exposure*	3768			4637		
Known prior exposure	15	1.56 (0.30)	0.30	75	-0.12 (1.10)	0.18
Vaccinated	1046	1.44 (0.33)	1.16	15479	0.34 (0.77)	0.80
**Variant amongst unvaccinated individuals with no known prior exposure**						
*Ref = Alpha (Oxford)*, *Delta (Northumbria)*	3526					
B.1.177	658	1.00 (2.15)	1.05			
Delta	645	-0.01 (1.01)	1.22	3310		
BA.1 Omicron	0			1327	-0.95 (2.07)	0.87
**Variant amongst vaccinated individuals**						
*Ref = Alpha (Oxford)*, *Delta (Northumbria)*	233					
B.1.177	3	1.82 (0.25)	0.22	0		
Delta	820	-1.37 (2.86)	1.13	6118		
BA.1 Omicron	0			9361	-0.59 (1.57)	0.83
**Variant amongst individuals with a known prior exposure**						
*Ref = Alpha (Oxford)*, *Delta (Northumbria)*	7					
B.1.177	3	5.17 (0.02)	0.57	0		
Delta	5	-3.05 (10.39)	0.20	10		
BA.1 Omicron	0			65	-0.35 (1.31)	0.18
**Vaccine product**						
*Ref = Pfizer/BioNTech BNT162b2*	2812			11506		
*AstraZeneca ChAdOx1 nCoV-19*	2017	-0.19 (1.16)	0.89	8685	-0.10 (1.08)	0.98
Not included in model						
**Vaccine dose amongst vaccinated individuals**						
*Ref = 1 dose*	527			1022		
≥2 doses	519	-0.09 (1.07)	0.73	14457	0.07 (0.95)	0.78
**Ethnicity**						
*Ref = White*	4290			18349		
All other ethnicities	539	0.24 (0.83)	0.32	1842	0.46 (0.70)	0.79
**Sex**						
*Ref = Female*	2445			10293		
Male	2384	-0.11 (1.09)	0.21	9898	0.50 (0.68)	0.50
**Health care worker**						
*Ref = no*	4646			19511		
Yes	183	0.44 (0.71)	0.31	680	0.28 (0.81)	0.31

### Among unvaccinated individuals with no known prior exposure, viral burden was higher among Alpha compared to B.1.177 infections

We defined unvaccinated individuals with no known prior exposure as those individuals who had neither a previous recorded infection, a previous positive test for spike antibodies, nor a vaccine at least 14 days prior. For samples sequenced at Oxford, Ct values in this group were higher for B.1.177 samples (Beta score = 1.00, VIP = 1.05) compared to Alpha. This corresponds to a 44% reduction in viral loads with Alpha. Among unvaccinated individuals with no known prior exposure, Ct values were similar among Alpha and Delta infections (Oxford: Beta score = -0.01, VIP = 1.22) and lower with BA.1 Omicron compared to Delta with borderline importance (Beta score = -0.95, VIP = 0.87).

### Among vaccinated individuals, viral burden was higher among Delta compared to Alpha infection

For individuals who were vaccinated or had a known prior exposure, we further categorised them according to whether they had either tested positive for spike antibodies prior to the first PCR-positive sample in the infection, or had 1 vaccine dose, or 2 or more vaccine doses. Individuals who had both a known prior exposure and were vaccinated were assigned to the appropriate vaccination group. Among the Oxford samples, Ct values were higher among vaccinated individuals, compared to those with no known prior exposure (Beta score = 1.44, VIP = 1.16), equating to a 67% reduction in viral burden with vaccination. Though the magnitude and importance of the signal was weaker–perhaps due to an increase in unidentified prior infections in the population–a similar pattern was observed among the Northumbria samples (Beta score = 0.34, VIP = 0.80). The impact of two or more vaccine doses over one on Ct values was not shown to be important (see Table 1) but the impact of variant among vaccinated individuals was important. Ct values were lower among Delta compared to Alpha infections (Oxford samples: Beta score = -1.37, VIP = 1.13), perhaps resulting from a greater genetic differences between the Delta variant (relative to the Alpha-variant) and the vaccine sequence. The measured effect corresponds to viral burden being 286% higher among Delta, compared to Alpha, infections. With borderline importance, we observed that Ct values were also lower among BA.1 Omicron infections, compared to Delta infections (Northumbria samples: Beta score = -0.59, VIP = 0.83) and were lower with the AstraZeneca ChAdOx1 nCoV-19 vaccine compared to Pfizer/BioNTech BNT162b2 (Oxford: Beta score = -0.19, VIP = 0.89; Northumbria samples: Beta score = -0.10, VIP = 0.98).

The number of samples with a described prior exposure (defined as an individual with a known prior infection or prior antibody positive result) was small (n = 90 across both data sets) and no clear effect was seen across the two dataset (see Table 1).

### The association between novel variants and higher viral burden is robust to assumptions about the shape of the within-host viral trajectory

**Fig 5:**
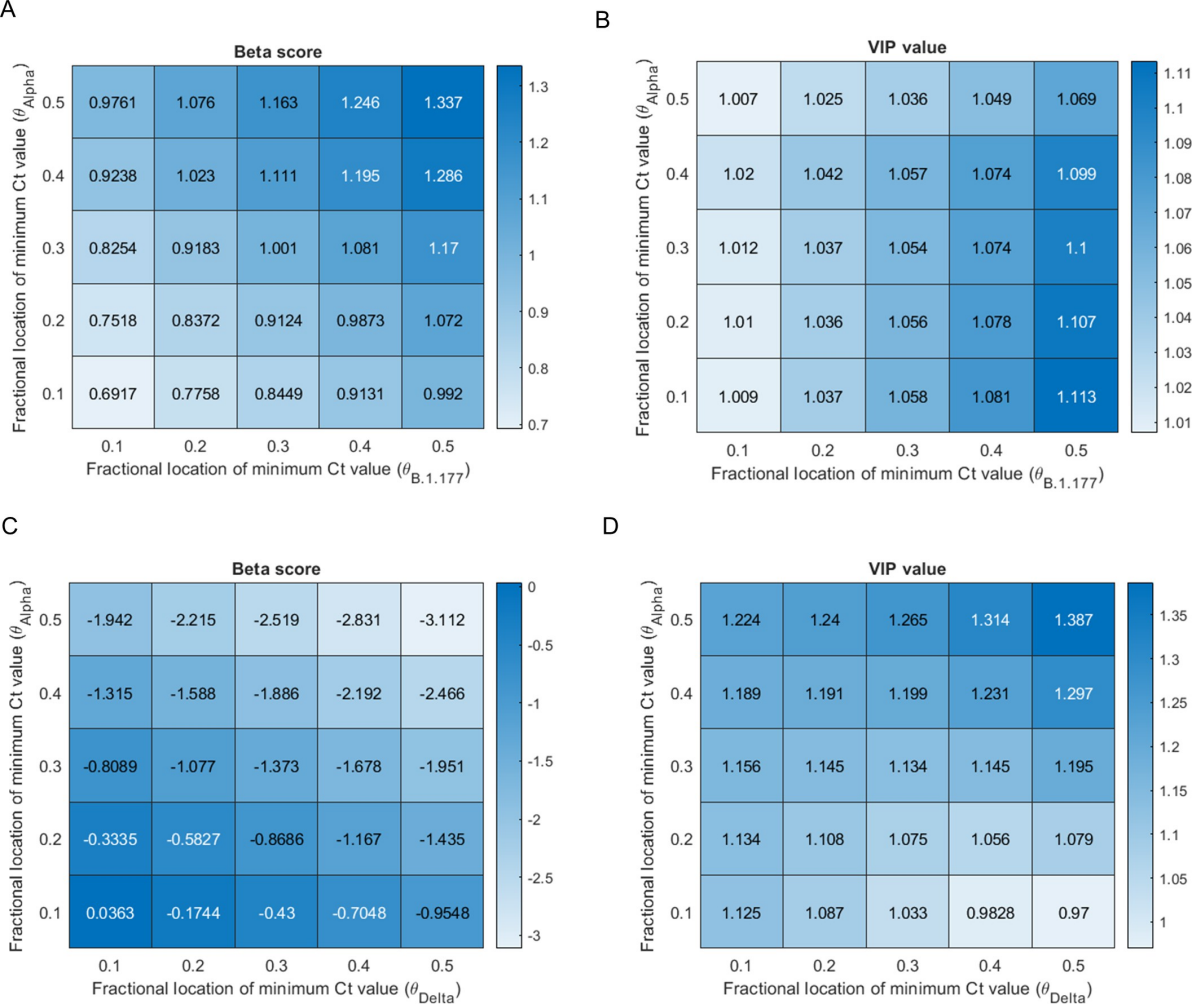
Sensitivity analysis investigating the impact of the shape of within-host viral trajectory on PLS regression analysis into the impact of variant on Ct values. Panels A) and C) show Beta scores, which can be considered to be equivalent to regression coefficients, defining the magnitude of the effect of the variant on the adjusted Ct values. Panels B) and D) show VIP values defining the importance of the association–where values greater than 1 are typically considered to indicate importance. Panels A) and B) investigate the association between the variant being B.1.177 (relative to Alpha) and Ct values among unvaccinated individuals with no known prior exposure. The Beta scores and VIP values vary with changes to the assumed asymmetry of the within-host viral burden trajectory associated with the B.1.177 lineage and the Alpha variant. The asymmetry is determined by changes to the fractional location of the minimum Ct value (peak viral burden) for each variant (*θ*_B.1.177_ and *θ*_Alpha_, respectively). Data sampled at Oxford. Panels C) and D) investigate the association between the variant being Delta (relative to Alpha) and Ct values among vaccinated individuals and how the Beta scores and VIP values vary with changes to *θ*_Alpha_ and *θ*_Delta_, respectively. Data from samples sequenced at Oxford.

Given our previous observation that the assumed asymmetry in the within-host viral burden trajectory can have a measurable impact on the adjusted Ct value, we conducted a sensitivity analysis on our PLS regression. We varied the parameter that determines the asymmetry of the within-host viral burden trajectory for each of the variants. Both the Beta score (Fig 5A) and VIP value (Fig 5B) for the indicator for the variant being B.1.177 rather than Alpha among unvaccinated individuals sampled at Oxford with no known prior exposure (i.e. no known prior infection nor prior antibody positive test) decreased as the assumed viral burden trajectories of B.1.177 were more skewed towards the start of the infection compared to Alpha (Fig 5A and 5B). These relationships are linked to the fact that although the Oxford samples span a large portion of the Alpha wave, they did not span the early part of the B.1.177 wave. It is noteworthy that the VIP value remained greater than unity across plausible parameter combinations, providing support for the observation that viral burden is higher in infections with the Alpha-variant compared to B.1.177, among unvaccinated individuals with no known prior exposure.

When evaluating the impact on Ct values of the variant being Delta (rather than Alpha) among vaccinated individuals (Fig 5C and 5D), both the VIP value and the magnitude of the Beta score increased as the assumed viral burden of Delta was more skewed towards the start of the infection compared to Alpha. These relationships are linked to the fact that the Oxford samples did not span the latter part of the Delta wave. The VIP value remained greater than unity (or very close to for higher discordance between the shape of the within-host trajectories of the two variants) across plausible parameter combinations. This analysis therefore provides support for the finding that samples with the Delta variant had a lower viral burden compared to samples with the Alpha variant among vaccinated individuals. In summary, our sensitivity analysis supports the finding that both the shift from B.1.177 to Alpha and the shift from Alpha to Delta was associated with an increase in viral burden amongst the population (mostly unvaccinated and mostly vaccinated, respectively) that prevailed at each stage.

## Discussion

We developed a framework to compare within-host viral burden across different SARS-CoV-2 variants from random survey data, such as the CIS. The method directly estimates the level of uncertainty in the time-since-infection of each sample due to the sparse nature of the sampling and the effect of differing epidemiological trends of SARS-CoV-2 variants. The method highlights how the combination of the within-host viral trajectory and the epidemiological trajectory of a viral variant can influence observed viral burden in survey data.

Using this framework, we inferred epidemiologically adjusted Ct values from samples sequenced as part of the CIS, a large-scale community survey, recruiting randomly selected private residential households and testing participants regardless of symptoms. Using partial least squares regression, we showed that in combination, factors included our analysis are, strongly associated with Ct values. We also assessed the magnitude and importance of the contribution of different variables to the response. Overall the findings indicate that viral burden shifts over time as population levels of immunity (notably from vaccination) change. In addition, for a given population-level immunological background, viral burden is influenced by viral variant. However, the effect sizes that we observed were modest in relation to the overall spread of the data.

We found that viral burden decreased over time and was higher among older individuals (13% and 17% higher for every 10 year age increase, in the samples sequenced in Oxford and Northumbria, respectively). Among individuals with no known prior immunity, viral burden was, on average, 115% higher among Alpha-variant compared to B.1.177 samples. Vaccination reduced viral burden, with the average reduction being 75% and 30% amongst the samples sequenced at Oxford and Northumbria, respectively. Among vaccinated individuals, there was also a pattern of viral burden increasing with the onset of a novel variant. Notably, viral burden was 286% higher among infections with the Delta-variant, compared to the Alpha-variant. We hypothesize that this finding could be linked to a greater genetic difference between the Delta variant (relative to the Alpha variant) and the vaccine strain.

A key question in virology is to understand how, and to what extent, evolution of a virus impacts its ability to transmit amongst a population. The degree to which evolution impacts within-host viral burden is one potential mechanisms. Although our study does not directly address this question, the effects sizes that we observe can be compared to data from a recent study by Lee et al. [35], in which SARS-CoV-2 infection probability among household contacts was investigated. That study found that on average, a Ct reduction of 15 (5 log increase in viral load) corresponded to a 160% increase in SARS-CoV-2 infection probability. This suggests that the effects sizes that we have observed in relation to viral variant and other variables, including age, are small. It is noteworthy, however, that Lee et al. [35], also showed that variables that we found to be associated with viral burden were associated with transmissibility. Notably, infections among children were less transmissible than those among adults, and infections with the Alpha variant were 50% more transmissible than those with the predecessor variant (B.1.177). In summary therefore, evidence suggests that variables such as age and viral variant impact transmissibility. Although viral burden differences may contribute to this effect, our data suggests that any effect is modest and that other factors must be at play. Such factors include differences between variants in the viral shedding rate, infectious period [36], or the per-virion probability of transmission.

For this study we determined viral variant from viral sequence data, which in practice meant excluding samples with a low viral burden. This is because only samples with Ct ≤30 are routinely sequenced, and additionally, samples with higher Ct values (a lower viral burden) are less likely to have sufficient genomic coverage to determine the variant. Although these restrictions could impact our qualitative estimates, we do not expect them to bias our main qualitative results. Furthermore, since individuals with a low viral burden contribute little to viral transmission [37], our study reflects the impact of viral variant and other variables on viral burden at levels that are relevant for transmission.

Monitoring the characteristics of novel SARS-CoV-2 variants will continue to be critical to public health decisions in the foreseeable future. As more countries roll out population representative surveys, accounting for epidemiological effects will remain important. More generally, any studies using community surveillance data that aim to consider traits that vary though infection (e.g. Ct values, immune markers), could be impacted by pathogen epidemiology and therefore could benefit from epidemiological adjustment. In summary, our study promotes a new way of critically analysing random survey data to acknowledge the combined impact of pathogen epidemiology and within-host traits that vary over the course of an infection. Although we have applied these methods to SARS-CoV-2 data, the methods are also applicable to the study of data relating to other pathogens.

## Methods

### Infection data processing

All individuals with at least one positive sample sequenced in Oxford or Northumbria, and with the virus assigned to one of the four major lineages as described above, were included in our analysis, and indexed *i = 1*…*n*, where *n* is the number of individuals. If an individual was infected by more than one major lineage during the study period, these were designated with an infection number *j*, where *j = 1* represents the first infection, *j = 2* the second infection, and so on. Positive samples were assumed to be part of the same infection if they were of the same major variant and were in a continuous sequence of positive samples (i.e. no negative intermediate samples). The index *k* denotes the *k*th sample of the infection. In the case of a non-continuous sequence of positive samples of the same major lineage, any additional positive samples were excluded from our study. Infections which were of the same major lineage but not in a continuous sequence of positive samples were excluded from the analysis. The list of variables used to describe the data are given in Table 2.

**Table 2:** Data used in the study.

Variable	Description
*t*_*ijk*_	Sample date of the *k*th sample of the *j*th infection of the *i*th individual
${\tilde{t}}_{ij}$	Sample date of the last negative before the first positive of the *j*th infection of the *i*th individual
*c*_*ijk*_	Observed Ct value of the *k*th sample of the *j*th infection of the *i*th individual.
*v*_*ij*_	Major variant of the *j*th infection of the *i*th individual
*φ*_*i*_	Sex of the *i*th individal
*e*_*i*_	Age group of the *i*th individual
*f*_*i*_	Vaccine product (AstraZeneca or Pfizer) of the first vaccine dose of the *i*th individual
$h_{i}^{r}$	Date of the *r*th vaccine dose of the *i*th individual

### Calculating epidemiologically adjusted Ct values

#### Step 1. Describing the within-host Ct trajectory

We assume that within-host Ct trajectories are piecewise linear and valley-shaped (Fig 1B), defined by the infected period (width, *w*) and the difference between the minimum Ct value and 40 (depth, *d*). Probability distributions for these variables (calculated in a discrete manner, each spaced by value 0.25 and 0.5 respectively) are derived from truncated discretised normal distributions, described by *p(d)* (Eq 1) and *p(w)* (Eq 2), with means $W_{v}^{mean}$ *and* $D_{v}^{mean}$ and standard deviations, *W*^*SD*^, *D*^*SD*^, so that

$$p(d)=(\Phi_{D}(d)-\Phi_{D}(d-0.5))/(\Phi_{D}(5)-\Phi_{D}(32))\;\mathrm{for}d=[5.5,6.0,6.5,\ldots ,32]$$ (1)

$$p(w)=\frac{\left(\Phi_{W}(w)-\Phi_{W}(w-0.25)\right)}{(\Phi_{W}(35)-\Phi_{W}(3))\;\mathrm{for}w}=[3.25,3.50,3.75,..,35]$$ (2)

where

$$\Phi_{D}(d)=normalCDF_{\left(D_{v}^{mean},D^{SD}\right)}(d)$$ (3)

$$\Phi_{W}(w)=normalCDF_{\left(W_{v}^{mean},W^{SD}\right)}(w)$$ (4)

The peak viral burden is assumed to occur at a time since infection equal to a fraction, *θ*_v_<1, of the total infected period. The parameters $W_{v}^{mean}$, *W*^*SD*^, *D*^*SD*^ and *θ*_v_ are derived from previous studies and varied in sensitivity analyses. The parameter $D_{v}^{mean}$, is iteratively inferred to a tolerance of 0.1 following implementation of the methodology described–which, for each sample, estimates an adjusted Ct value–and calculated to equal twice the difference between 40 and the mean adjusted Ct value for that variant. For ease of reference, all other variables described here and throughout the following derivation are listed in Table 3.

**Table 3:** Description of additional variables and parameters used in calculation of adjusted Ct values.

Variable	Description	
a	Time since infection (discrete: each unit equivalent to 0.25 days)	
*d*	Minimum Ct -40 (viral trajectory depth)	
*w*	Infected period (viral trajectory width) (days)	
*v*	Variant	
*τ*	Time step (discrete: each unit equivalent to 0.25 days)	
*a*_*ijk*_	Time since infection of the *k*th sample of the *j*th infection of the *i*th individual (days) (discrete: each unit equivalent to 0.25 days)	
*u*_*a*,*τ*,*v*_	Estimated of number of people with time since infection*a* at time step *τ*, with variant *v*	
*r*_*τ*,*v*_	The proportion of incident infections during time step *τ* that are of variant *v*	
*I*_*τ*_	Number of new infections (incidence) during time step *τ*	
*A*_*d*,*w*,*θ*_ *(C)*	Time since infection at Ct value, C, during the down phase of the assumed valley shaped Ct trajectory	
${\tilde{A}}_{d,w,\theta }(C)$	Time since infection at the Ct value, C, during the up phase of the assumed valley shaped Ct trajectory	
${\tilde{c}}_{ijk}$	Adjusted Ct value of the *k*th sample of the *j*th infection of the *i*th individual	
*F*_*sample_ijk_*_(*C*)	Cumulative probability for the expected Ct value, *C* for sample *ijk*	
*F*_*flat*_(*C*)	Cumulative probability for the expected Ct value, C, assuming a flat trajectory (constant incidence)	
**Parameters**	**Description**	**Values**
θ_*v*_	Fractional location of the minimal Ct across the infected period, with variant *v*	0.3
$W_{v}^{mean}$	Mean viral trajectory width (infected period, days)	10.1
$D_{v}^{mean}$	Mean viral trajectory depth (difference between minimum Ct value and 40)	Iteratively inferred to equal 10 + 2(30−mean adj Ct) with initial condition: Ct = 20.
*W* ^*SD*^	Standard deviation of viral trajectory width	5
*D* ^*SD*^	Standard deviation of viral trajectory depth	1.7

#### Step 2. Estimating the distribution of time since infection for different SARS-CoV-2 variants over calendar time

We estimated the distribution of infections in the population stratified by variant and time since infection over calendar time using published estimates of total incidence of SARS-CoV-2 in the UK (www.ons.gov.uk/peoplepopulationandcommunity/healthandsocialcare/conditionsanddiseases/datasets/coronaviruscovid19infectionsurveydata) and published estimates of the proportion of incident infections with each of the major variants under study (B.1.177, Alpha, Delta and BA.1 Omicron) over time from the COVID-19 Genomics UK Consortium (COG-UK: www.cogconsortium.uk). Working in discrete time steps (τ = 1,2,3…) that are 0.25 days each, we define *I*_*τ*_ to be the incidence during time step, *τ* and *r*_*τ*,*v*_ to be the proportion of incident infections during time step *τ* that are of variant *v* (*v* = 1:4 represent B.1.177, Alpha, Delta and BA.1 Omicron, respectively). We further define *u*_*a*,*τ*,*v*_ to be the number of infections with time since infection, *a* (stratified as discrete time steps of 0.25 days each), during time step *τ* with variant *v*. The number of incident infections (i.e. infections with time since infection = 0) during time step *τ* with each variant *v* is estimated to be the product of the total incidence during that time step and the fraction of incident infections of that variant (*u*_0,*τ*,*v*_ = *r*_*τ*,*v*_*I*_*τ*_). To estimate *u*_*a*,*τ*,*v*_ for each *a>0*, we assume that the infected periods are taken from a truncated normal distribution with mean, $W_{v}^{mean}$, and variance *W*^*SD*^_._ Therefore, the number of infections of time since infection *a*, at time step *τ* is calculated to be the number of incident infections from time step *τ-a* that are still persisting after a time *a*, thus:

$$u_{a,\tau ,v}=u_{0,\tau -a,v}(1-normalCDF_{\left(W_{v}^{mean},W^{SD}\right)}(a)).$$ (5)

#### Step 3. For each sample and each infected period, estimate a time since infection distribution

For each sample and for each assumed infected period (*w*), we inferred the distribution of time since infection. We first selected the distribution (Step 2) that corresponds to the sample date and variant of the sample and adjusted it to account for known bounds on the time since infection for that sample, measured in days. The bounds ($a_{ij}^{ma{\tilde{x_{ijk}}}_{ij}}$ and $a_{ij}^{mi{n_{ijk}}_{ij,k-1}}$) are derived by considering information on Ct values at previous samples and scaled to account for the transformation to discrete time steps. The time since infection probability distribution for each sample is then given by:

$$p(a_{ijk}|w,t_{ijk},v_{ij})=\{0\;if\;a_{ijk}\{\begin{array}{l} >w\;or\\ >a_{ij}^{max\;\mathrm{or}}\\ <a_{ij}^{min}\{ \end{array}|\frac{u_{a_{ijk},4t_{ijk},v_{ij}}}{\sum_{a=4a_{ij}^{min}}^{4\;\min(a_{ij}^{max}\sum }u_{a,4t_{ijk},v_{ij}}\;\mathrm{otherwise}}\{$$ (6)

#### Step 4. Infer a sample-specific expected distribution of Ct values

For each sample, based upon the sample time (*t*_*ijk*_) and variant (*v*_*ij*_), we derived an expected distribution of Ct values (Eq 7). This was done by conditioning on the time since infection (*a*) and the depth (*d*) and width (*w*) of the within host viral trajectory. These conditional probabilities were combined with the time since infection distributions derived in step 3 and the within-host parameter distributions described in step 1.

$$p(C-0.5\leq c<C|t_{ijk},v_{ij})=\frac{\sum_{a_{ijk}}\sum_{d}\sum_{w}p(C-0.5\leq c<C|a_{ijk},d,w)p(a_{ijk},d,w|t_{ijk},v_{ij})}{\sum_{c=0.5}^{40}\sum_{a_{ijk}}\sum_{d}\sum_{w}p(C-0.5\leq c<C|a_{ijk},d,w)p(a_{ijk},d,w|t_{ijk},v_{ij})}$$ (7)

where the probability of a particular time since infection (*a*_*ijk*_), trajectory width (*w*) and trajectory depth (*d*) is given by:

$$p(a_{ijk},d,w|t_{ijk},v_{ij})=p(a_{ijk}|w,t_{ijk},v_{ij})p(d)p(w)$$ (8)

and the probability of the Ct value (*c*) falling within a certain discrete boundary, given the time since infection and the width and depth of the viral trajectory, is defined as 1 or 0 depending upon whether it matches up with the valley-shaped viral trajectory curve (Fig 1B), as shown below:

$$p(C-0.5\leq c<C|a_{ijk},w,d)=\left\{ \begin{array}{l} 1\;if\;A_{d,w,\theta_{v}}(C-0.5)<a_{ijk}<A_{d,w,\theta_{v}}(C)\;and\;a_{ijk}\leq \theta w\\ 1\;if\;{\tilde{A}}_{d,w,\theta_{v}}(C-0.5)<a_{ijk}<{\tilde{A}}_{d,w,\theta_{v}}(C)\;and\;a_{ijk}>\theta w\\ 0\;otherwise \end{array}\right.$$ (9)

Where C is a dummy variable representing the Ct value, and

$$A_{d,w,\theta_{v}}(C)=\frac{(40-C)\theta_{v}w}{d}$$ (10)

and

$${\tilde{A}}_{d,w,\theta_{v}}(C)=w-\frac{(40-C)(1-\theta_{v})w}{d}$$ (11)

are dummy variables that describe the relationship between the Ct value (C) and the time since infection ($A_{d,w,\theta_{v}}(C)$ and ${\tilde{A}}_{d,w,\theta_{v}}(C)$), during down the phase and up phase of the valley-shaped trajectory, respectively.

#### Step 5. Calculate an expected distribution of Ct values for a flat epidemic trajectory

The full process for calculating an expected distribution of Ct values (steps 1–4) was repeated under an assumption of a flat epidemic trajectory (constant incidence) rather than a variant-specific trajectory.

#### Step 6. For each sample, infer an epidemiologically adjusted Ct value

For each sample, we identified the percentile that the observed Ct (*c*_*ijk*_) falls in, among the sample-specific expected Ct distribution. The adjusted Ct value (${\tilde{c}}_{ijk}$) was then derived by identifying the Ct value at that percentile within the expected distribution of Ct values based upon a flat epidemic trajectory (Fig 1C).

$${\tilde{c}}_{ijk}=F_{flat}^{-1}(F_{sample_{ijk}}(c_{ijk}))$$ (12)

where

$$F_{sample_{ijk}}(\hat{C})=p(\hat{c}<\hat{C}|sample_{ijk})=\sum_{C=5.5,6.0,\ldots }^{\hat{C}}p(C-0.5\leq c<C|t_{ijk},v_{ij})$$ (13)

$$F_{flat}(\hat{C})=p(\hat{c}<\hat{C}|\mathrm{flat}\;\mathrm{epidemic})=\sum_{C=5.5,6.0,\ldots }^{\hat{C}}p(C-0.5\leq c<C|\mathrm{flat}\;\mathrm{epidemic})$$ (14)

### Implementation of analysis

All analyses were implemented in Matlab and the code is available at https://github.com/helenfryer1000000/epidemiologically-adjusted-viral-load. Estimation of adjusted Ct values was implemented using a bespoke script. Partial least squares regression was implemented using the PLSregress function, which is part of the Statistics and Machine Learning toolbox in Matlab. Quantile median regression was implemented using the function qr_standard, provided at: https://github.com/zjph602xtc/Quantile_reg.

## Supporting information

S1 Table A review of published studies investigating the impact of viral variant on Ct values. (DOCX)

S2 Table Variance inflation factor (VIF) values. (DOCX)

S1 Fig Mean squared error and loading plots relating to the partial least squares regression analysis. (TIF)

S2 Fig Plots showing the distribution of the data provided in Fig 2. (TIF)

S1 Data A list of accession number for samples included in this study. Sequences can be accessed via the the European Nucleotide Archive (ENA) at https://www.ebi.ac.uk/ena/browser/home.
(TXT)

S1 Text A list of members of the COVID-19 Genomics UK (COG-UK) consortium. (DOCX)
