# Correction: Viral burden is associated with age, vaccination, and viral variant in a population-representative study of SARS-CoV-2 that accounts for time-since-infection-related sampling bias

**DOI:** 10.1371/journal.ppat.1011706

**Published:** 2023-10-06

**Authors:** Helen R. Fryer, Tanya Golubchik, Matthew Hall, Christophe Fraser, Robert Hinch, Luca Ferretti, Laura Thomson, Anel Nurtay, Lorenzo Pellis, Thomas House, George MacIntyre-Cockett, Amy Trebes, David Buck, Paolo Piazza, Angie Green, Lorne J Lonie, Darren Smith, Matthew Bashton, Matthew Crown, Andrew Nelson, Clare M. McCann, Mohammed Adnan Tariq, Claire J. Elstob, Rui Nunes Dos Santos, Zack Richards, Xin Xhang, Joseph Hawley, Mark R. Lee, Priscilla Carrillo-Barragan, Isobel Chapman, Sarah Harthern-Flint, The COVID-19 Genomics UK (COG-UK) consortium, David Bonsall, Katrina A. Lythgoe

In the Calculating epidemiologically adjusted Ct values subsection of the Methods, there are a number of formatting errors in the sixth and ninth equations. Please view the complete, correct sixth equation here:

$$p(a_{ijk}|w,t_{ijk},v_{ij})=\{\begin{array}{l} \;0\;\mathrm{if}\;a_{ijk}\left\{ \begin{array}{l} >w\;\mathrm{or}\\ >a_{ij}^{\max}\;\mathrm{or}\\ <a_{ij}^{\min} \end{array}\right.\\ u_{a_{ijk},4t_{ijk},v_{ij}}/\sum_{a=4a_{ij}^{\min}}^{4\min(a_{ij}^{\max},w)}u_{a,4t_{ijk},v_{ij}}\;\mathrm{otherwise} \end{array}$$

Please also view the complete, correct ninth equation here:

$$p(C-0.5\leq c<C|a_{ijk},w,d)=\{\begin{array}{l} 1\;\mathrm{if}\;A_{d,w,\theta_{v}}(C-0.5)<a_{ijk}<A_{d,w,\theta_{v}}(C)\;\mathrm{and}\;a_{ijk}\leq \theta_{v}w\\ 1\;\mathrm{if}\;{\tilde{A}}_{d,w,\theta_{v}}(C-0.5)<a_{ijk}<{\tilde{A}}_{d,w,\theta_{v}}(C)\;\mathrm{and}\;a_{ijk}>\theta_{v}w\\ 0\;\mathrm{otherwise} \end{array}$$

The in-text expressions on page 20, line 5 are incorrect. The correct expressions are:

$$a_{ij}^{\max}=4(t_{ijk}-{\tilde{t}}_{ij})\;\mathrm{and}\;a_{ij}^{\min}=4(t_{ijk}-t_{ij,k-1})$$

There are a number of errors in the caption for Fig 4, “Adjusted Ct values plotted against different factors”. Please see the complete, correct Fig 4 caption here.

**Fig 4:**
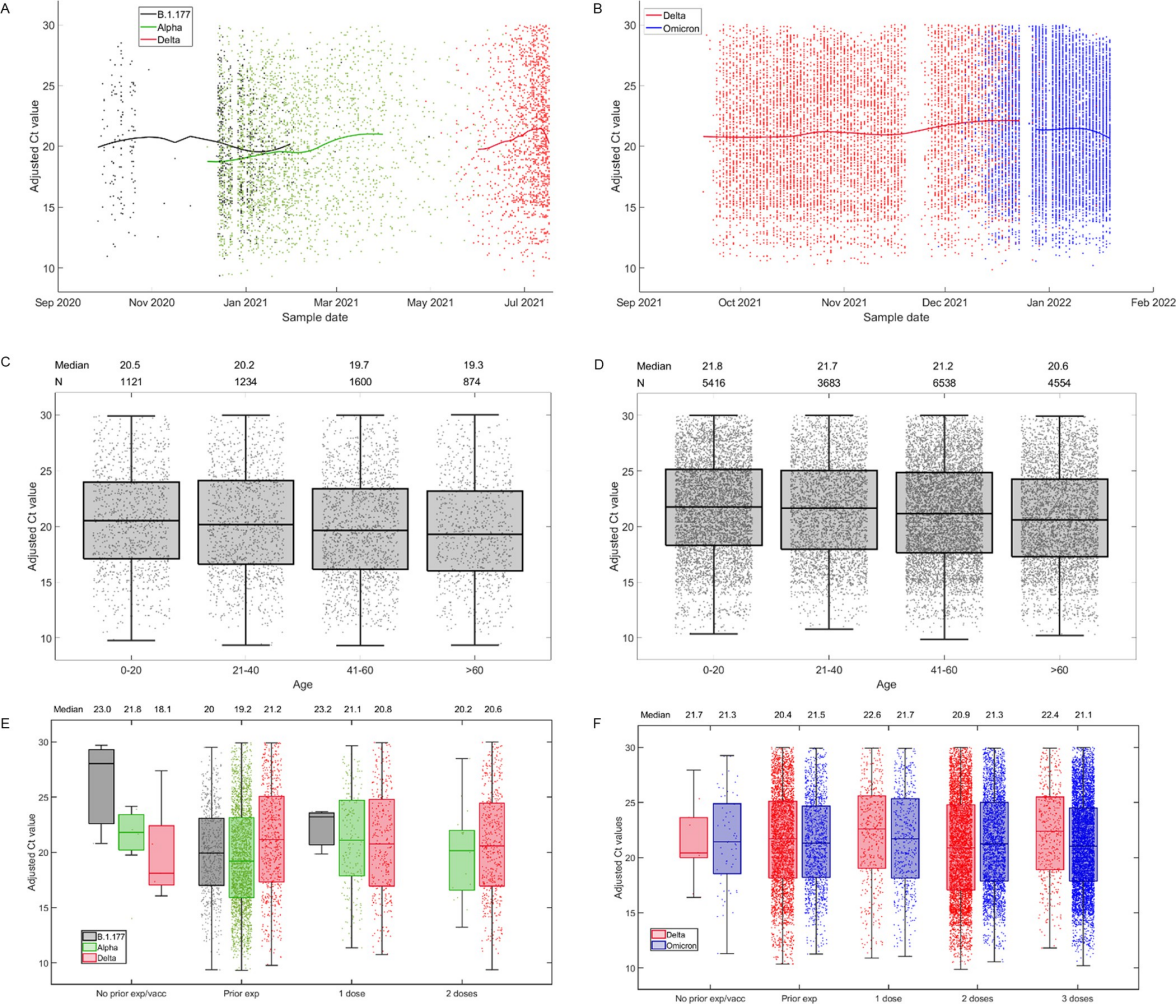
Adjusted Ct values plotted against different factors. For samples sequenced at Oxford (A, C and E) and at Northumbria (B, D and F), adjusted Ct values are plotted against different variables. Panel A) and B) show a LOESS fit (smoothing parameter = 0.55) of adjusted Ct values over sample date, categorised by variant. Panels C) and D) show box and whisker plots of adjusted Ct values by age category. Panels E) and F) show box and whisker plots of adjusted Ct values by prior vaccination and/or infection, by variant. Horizontal lines represent the median and interquartile range. Parameter values used in these calculations are listed in Table 3.

In the Investigating variables associated with within-host viral burden subsection of the Results, there is an error on the third line of the second paragraph referencing Fig 4C and Fig 4F. The correct reference is Fig 4E and Fig 4F.
