# Supplementary material for: Viral burden is associated with age, vaccination, and viral variant in a population-representative study of SARS-CoV-2 that accounts for time-since-infection-related sampling bias
Source: PLoS Pathog. 2023 Aug 14;19(8):e1011461. doi: 10.1371/journal.ppat.1011461 (PMC10449197; doi:10.1371/journal.ppat.1011461)
Supplement: S1 Table — (DOCX) [file ppat.1011461.s001.docx]

| **Reference** | **Ct values** | | | | **How were individuals chosen to be part of the study** | **Longitudinal?** | **How was strain determined?** |
| --- | --- | --- | --- | --- | --- | --- | --- |
|  | **Predecessor variants to B.1.17** | **B.1.1.7** | **B.1.351** | **Delta** |  |  |  |
| Frampton [1] | Ct Mean=32  SD=4.8  N=143 | Mean Ct=28.8  SD=4.7  N=341 |  |  | Individuals acutely admitted to hospital in London. | No | S-gene target failure |
| Calistri [2] | Median Ct=16.9  95% CI=[10.4,19.9]  N=965 | Median Ct=15.8  95% CI=[9.6,19.6]  N=313  p-value<10^-4^ (relative to predecessor) |  |  | Swabs from three provinces of Abruzzo in Italy were collected based on clinical symptoms or reported contact with confirmed COVID-19 cases. | Yes, but only from some individuals. Infected periods were calculated from only those individuals with 2 or more positive samples. | Whole genome sequencing |
| Kidd [3] | median Ct (ORF1ab) =22.30  median Ct (N gene)=23.1  N=450 | median Ct (ORF1ab)=18.16  median Ct (N gene)=19.39  N=178  p-value<10^-5^ (relative to predecessor for both ORF1ab and N gene) |  |  | Samples in the UK Department of Health and Social Care Test and Trace network. | No | S-gene target failure |
| Cosentino [4] | Ct at self reported symptom onset=22.7  95% CI=[22.4,23.0]  N=3272 | Ct at self reported symptom onset= 21.3  95% CI=[21.1,21.6]  N=11496  p-value<10^-6^ (relative to predecessor) | Ct at self reported symptom onset= 21.6 95% CI=[21.1,22.0]  N=1366  p-value<10^-6^ (relative to predecessor) |  | Community testing. Symptomatic individuals | Principally no, although 20% of individuals had two or more samples | S-gene target failure |
| Kissler [5] | Peak viral concentration: Ct=20.1  95% CI=[18.3,21.7]  N=41 | Peak viral concentration Ct=21.0  95% CI=[19.1,20.9]  N=36  No meaningful difference compared to predecessor reported |  | Peak viral concentration  Ct=19.8  95% CI=[18.0,22.0]  N=36  No meaningful difference compared to predecessor reported | Individuals associated with a professional basketball league | Yes (testing done daily) | Whole genome sequencing |
| Ke [6] | Predicted minimum Ct (in saliva)= 23.7  N=44 | Predicted minimum Ct (in saliva)= 24.2  N=16  p-value=0.32 (relative to predecessor) |  |  | Positive samples, or contacts of positive samples, from twice weekly testing of all faculty, staff and students at a university campus. | Yes (daily testing for up to 14 days | Whole genome sequencing |
| Pouwels [7] |  | Median Ct=31.6 *  IQR=[22.8,33.7]  N=577  *in new PCR positives  vaccinated for 14  ≥21 days after dose 1 or  <14 days after dose 2 |  | Median Ct=30.1  IQR=[18.6,33.7]  N=110  in new PCR-positives vaccinated for 14  ≥21 days after dose 1 or  <14 days after dose 2 | Population representative survey (includes asymptomatic) | Yes, although samples are typically spaced 1 week or 1 month apart, meaning that most recorded infections have only one positive sample | Estimated based upon sample date |
| Li [8] | Median Ct =34.31  IQR=[31-36]  N=63 |  |  | Median Ct =24  IQR=[19-29]  N=62 | Close contacts of confirmed cases | Yes (daily testing) | Whole genome sequencing |

**S1 Table. A review of published studies investigating the impact of viral variant on Ct values.**

**References for Table 1.**

1. Frampton D, Rampling T, Cross A, Bailey H, Heaney J, Byott M, et al. Genomic characteristics and clinical effect of the emergent SARS-CoV-2 B.1.1.7 lineage in London, UK: a whole-genome sequencing and hospital-based cohort study. Lancet Infect Dis. 2021;21(9):1246-56. Epub 2021/04/16. doi: 10.1016/S1473-3099(21)00170-5. PubMed PMID: 33857406; PubMed Central PMCID: PMCPMC8041359 manufacture of UCL-Ventura continuous positive airway pressure device for patients with COVID-19 from the UK Department of Health and Social Care, during the conduct of the study; grants and advisory board fees paid to institution research fund from NewB; grants from DSTL; advisory board and speaking fees paid into institutional research fund from Amormed, Biotest, General ElectricBaxter, Baxter, Roche, Bayer, and Shionogi; and grants from Critical Pressure and Apollo Therapeutics, outside the submitted work. All other authors declare no competing interests.

2. Calistri P, Amato L, Puglia I, Cito F, Di Giuseppe A, Danzetta ML, et al. Infection sustained by lineage B.1.1.7 of SARS-CoV-2 is characterised by longer persistence and higher viral RNA loads in nasopharyngeal swabs. Int J Infect Dis. 2021;105:753-5. Epub 2021/03/09. doi: 10.1016/j.ijid.2021.03.005. PubMed PMID: 33684558; PubMed Central PMCID: PMCPMC7934691.

3. Kidd M, Richter A, Best A, Cumley N, Mirza J, Percival B, et al. S-Variant SARS-CoV-2 Lineage B1.1.7 Is Associated With Significantly Higher Viral Load in Samples Tested by TaqPath Polymerase Chain Reaction. J Infect Dis. 2021;223(10):1666-70. Epub 2021/02/14. doi: 10.1093/infdis/jiab082. PubMed PMID: 33580259; PubMed Central PMCID: PMCPMC7928763.

4. Cosentino G, Bernard M, Ambroise J, Giannoli JM, Guedj J, Debarre F, et al. SARS-CoV-2 viral dynamics in infections with Alpha and Beta variants of concern in the French community. J Infect. 2022;84(1):94-118. Epub 2021/07/31. doi: 10.1016/j.jinf.2021.07.031. PubMed PMID: 34329672.

5. Kissler SM, Fauver JR, Mack C, Tai CG, Breban MI, Watkins AE, et al. Viral Dynamics of SARS-CoV-2 Variants in Vaccinated and Unvaccinated Persons. N Engl J Med. 2021;385(26):2489-91. Epub 2021/12/24. doi: 10.1056/NEJMc2102507. PubMed PMID: 34941024; PubMed Central PMCID: PMCPMC8693673.

6. Ke R, Martinez PP, Smith RL, Gibson LL, Mirza A, Conte M, et al. Daily longitudinal sampling of SARS-CoV-2 infection reveals substantial heterogeneity in infectiousness. Nat Microbiol. 2022;7(5):640-52. Epub 2022/04/29. doi: 10.1038/s41564-022-01105-z. PubMed PMID: 35484231.

7. Pouwels KB, Pritchard E, Matthews PC, Stoesser N, Eyre DW, Vihta KD, et al. Effect of Delta variant on viral burden and vaccine effectiveness against new SARS-CoV-2 infections in the UK. Nat Med. 2021;27(12):2127-35. Epub 2021/10/16. doi: 10.1038/s41591-021-01548-7. PubMed PMID: 34650248; PubMed Central PMCID: PMCPMC8674129.

8. Li B, Deng A, Li K, Hu Y, Li Z, Shi Y, et al. Viral infection and transmission in a large, well-traced outbreak caused by the SARS-CoV-2 Delta variant. Nat Commun. 2022;13(1):460. Epub 2022/01/26. doi: 10.1038/s41467-022-28089-y. PubMed PMID: 35075154; PubMed Central PMCID: PMCPMC8786931.
