# Supplementary material for: Viral burden is associated with age, vaccination, and viral variant in a population-representative study of SARS-CoV-2 that accounts for time-since-infection-related sampling bias
Source: PLoS Pathog. 2023 Aug 14;19(8):e1011461. doi: 10.1371/journal.ppat.1011461 (PMC10449197; doi:10.1371/journal.ppat.1011461)
Supplement: S2 Table — (DOCX) [file ppat.1011461.s002.docx]

|  | Variance inflation factor (VIF) | |
| --- | --- | --- |
|  | Oxford | Northumbria |
| **Sample date** | 7.9 | 3.3 |
| **Age** | 1.7 | 2.4 |
| **Prior exposure** |  |  |
| *Ref = Unvaccinated with no known prior exposure* |  |  |
| Known prior exposure | 2.1 | 7.5 |
| Vaccinated | 4.9 | 5.0 |
| **Variant amongst unvaccinated individuals with no known prior exposure** |  |  |
| *Ref = Alpha (Oxford), Delta (Northumbria)* |  |  |
| B.1.177 | 1.4 |  |
| Delta | 4.3 |  |
| BA.1 Omicron |  | 2.0 |
| **Variant amongst individuals with a known prior exposure** |  |  |
| *Ref = Alpha (Oxford), Delta (Northumbria)* |  |  |
| B.1.177 | 1.4 |  |
| Delta | 1.7 |  |
| BA.1 Omicron |  | 7.5 |
| **Variant amongst vaccinated individuals** |  |  |
| *Ref = Alpha (Oxford),*  *Delta (Northumbria)* |  |  |
| B.1.177 | 1.0 |  |
| Delta | 6.5 |  |
| BA.1 Omicron |  | 3.4 |
| **Vaccine dose amongst vaccinated individuals** |  |  |
| *Ref = 1 dose* |  |  |
| ≥2 doses | 2.3 | 5.3 |
| **Vaccine product** |  |  |
| *Ref = Pfizer/BioNTech BNT162b2* |  |  |
| *AstraZeneca ChAdOx1 nCoV-19* | 1.5 | 1.5 |
| **Ethnicity** |  |  |
| *Ref=White* |  |  |
| All other ethnicities | 1.1 | 1.1 |
| **Sex** |  |  |
| *Ref = Female* |  |  |
| Male | 1.0 | 1.0 |
| **Health care worker** |  |  |
| *Ref=no* |  |  |
| Yes | 1.0 | 1.1 |

**S2 Table. Variance inflation factor (VIF) values.**
