# Supplementary figures and images for: Viral burden is associated with age, vaccination, and viral variant in a population-representative study of SARS-CoV-2 that accounts for time-since-infection-related sampling bias
Source: PLoS Pathog. 2023 Aug 14;19(8):e1011461. doi: 10.1371/journal.ppat.1011461 (PMC10449197; doi:10.1371/journal.ppat.1011461)

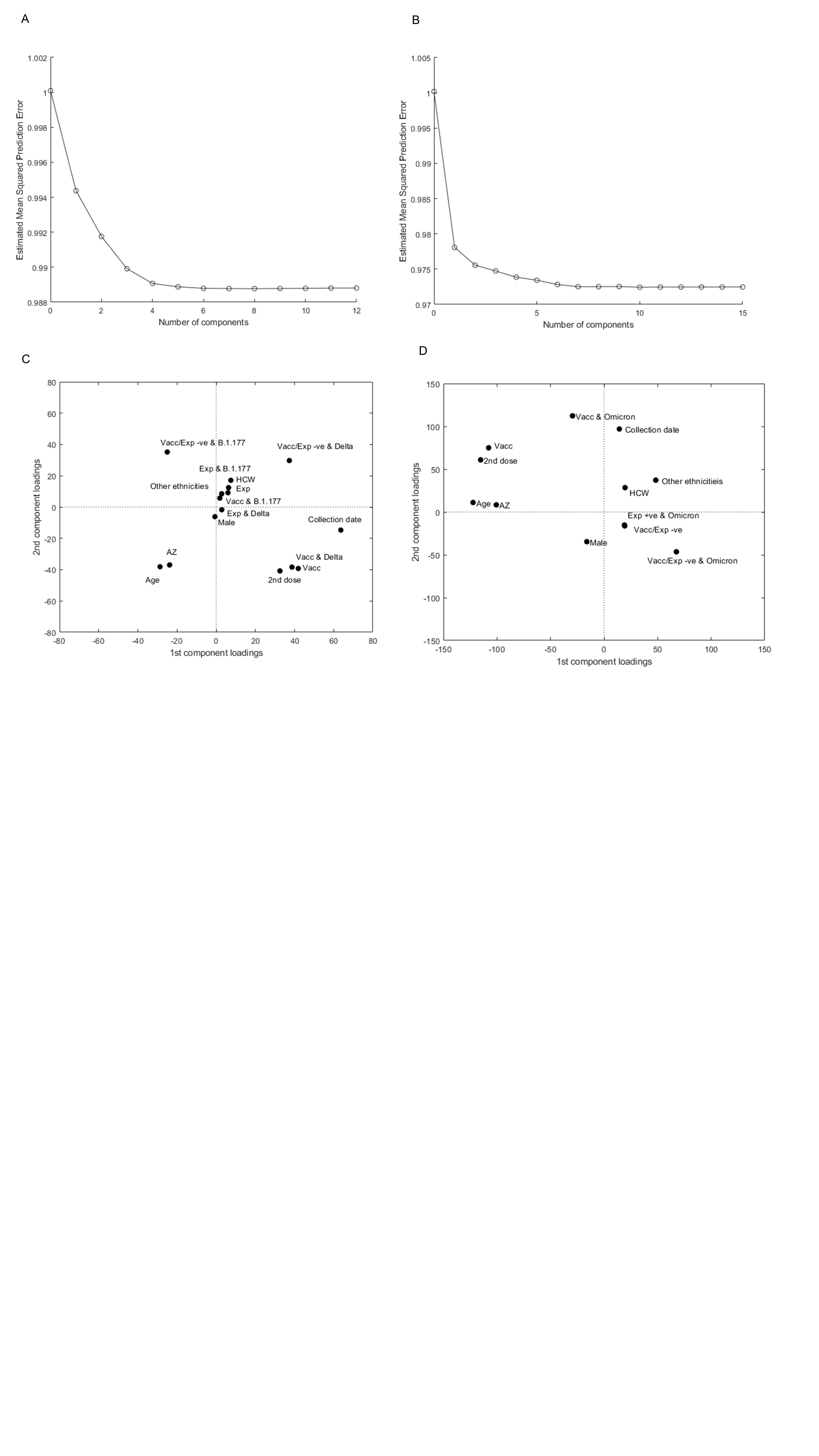

Supplement: S1 Fig — (TIF) [file ppat.1011461.s003.tif]

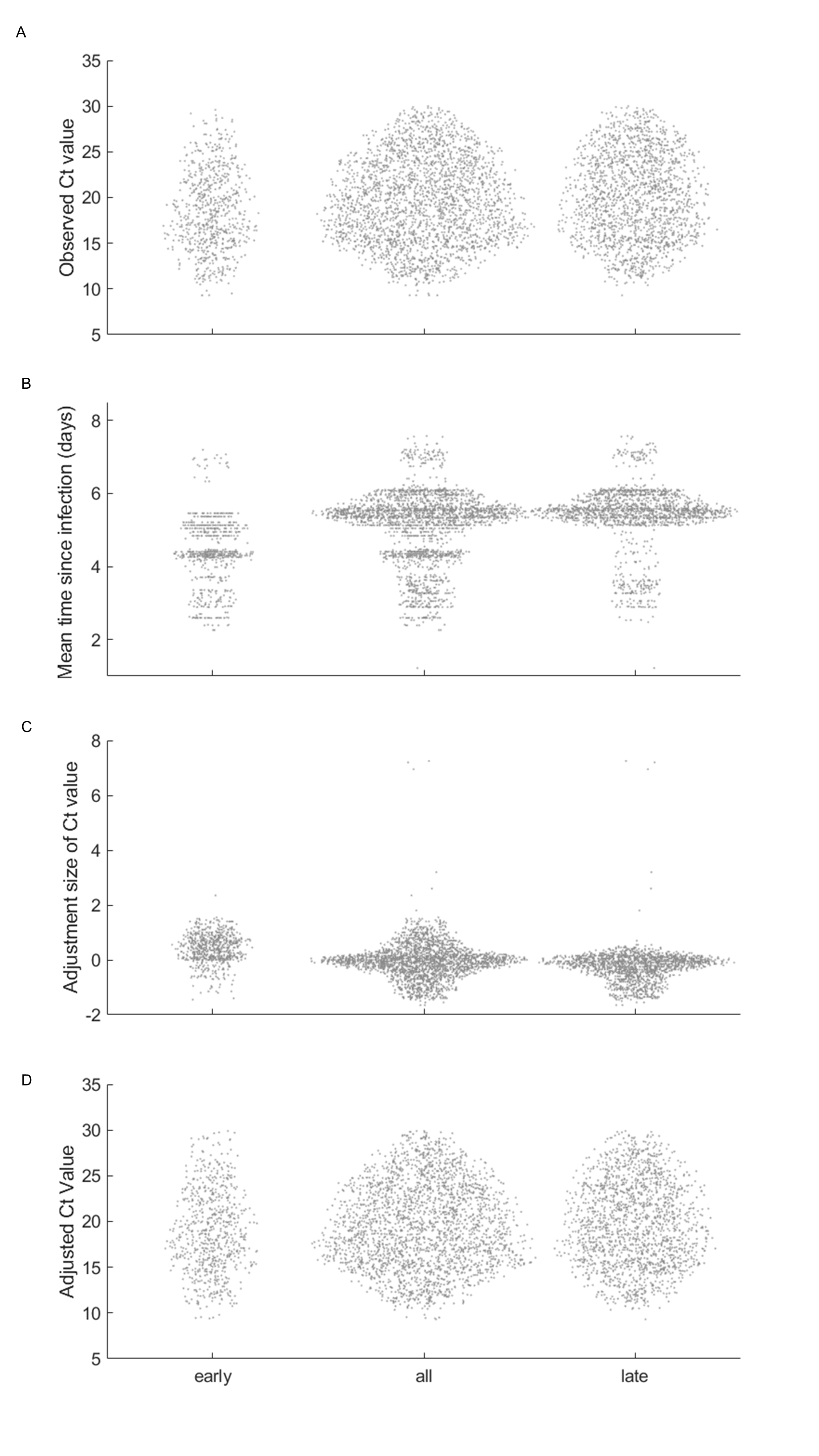

Supplement: S2 Fig — (TIF) [file ppat.1011461.s004.tif]
